# Supplementary material for: Spatiotemporal patterns and entomological predictors of dengue transmission in Urban Surat, India (2016–2020): A surveillance-based risk modelling study
Source: PLOS Glob Public Health. 2026 Mar 19;6(3):e0006086. doi: 10.1371/journal.pgph.0006086 (PMC13001960; doi:10.1371/journal.pgph.0006086)
Supplement: S1 Text — Table B. Zonal distribution of laboratory-confirmed reported Dengue incidences, notification rates, population characteristics, entomological indices, and reporting sectors in Surat, India, 2016–2020. (DOCX) [file pgph.0006086.s001.docx]

**Table A in S1 Text. Annual Dengue Case Notification Rates and Entomological Indices in Surat, India (2016–2020)**

| **Year** | **Mid-Year Population Estimate** | **Total Cases (N, per 100,000)** | **Public Sector (N, per 100,000)** | **Private Sector (N, per 100,000)** | ***House Index >1 (%) **** | **Container Index >10(%) **** | **Breteau Index >5 (%) ***** |
| --- | --- | --- | --- | --- | --- | --- | --- |
| 2016 | 5,566,112 | 619 (11.1) | 449 (8.1) | 170 (3.1) | 171 (27.6%) | 0 | 0 (0.0%) |
| 2017 | 5,816,701 | 272 (4.7) | 230 (3.9) | 42 (0.7) | 127 (46.7%) | 0 | 7 (2.6%) |
| 2018 | 6,078,457 | 249 (4.1) | 221 (3.6) | 28 (0.5) | 103 (41.4%) | 0 | 2 (0.8%) |
| 2019 | 6,352,273 | 375 (5.9) | 366 (5.8) | 9 (0.1) | 150 (40.9%) | 0 | 4 (1.1%) |
| 2020 | 6,637,703 | 143 (2.2) | 143 (2.2) | 0 (0.0) | 42 (30.2%) | 0 | 1 (0.7%) |
| **Total** | **30,451,246** | **1,658 (5.44)** | **1,409 (4.62)** | **249 (0.81)** | **593 (36.0%)** * | **0** | **14 (0.9%)** ** |

**Statistical tests**:
* House Index (HI): χ²(4, N = 1646) = 41.2, *p* < 0.001
** Breteau Index (BI): χ²(4, N = 1646) = 15.2, *p* = 0.004

**Note**: Case notification rates are expressed per 100,000 mid-year population. Entomological indices reflect the percentage of dengue cases each year associated with House Index >1%, Container Index >10%, and Breteau Index >5%.

**Table B in S1 Text. Zonal Distribution of Dengue Cases, Notification Rates, Population Characteristics, Entomological Indices, and Reporting Sectors in Surat (2016–2020)**

| **Zone** | **Central** | **East** | **North** | **South-East** | **South-West** | **South** | **West** | **Total** |
| --- | --- | --- | --- | --- | --- | --- | --- | --- |
| **Notified Cases N (% of Total)** | 151 (9.1%) | 267 (16.1%) | 164 (9.9%) | 416 (25.1%) | 106 (6.4%) | 374 (22.6%) | 180 (10.9%) | **1,658 (100%)** |
| **Notification Rate** (per 100,000) | 36.9 | 22.5 | 22.9 | 55.1 | 30.4 | 40.1 | 40.0 | — |
| **Census 2011 Population** | 408,760 | 1,186,950 | 716,110 | 754,128 | 348,423 | 781,070 | 449,943 | — |
| **Population Density** (per km²) | 49,971 | 30,303 | 19,392 | 38,390 | 3,105 | 11,253 | 8,288 | — |
| ***House Index >1 (%)* *** | 50 (33.3%) | 68 (25.6%) | 52 (31.7%) | 198 (48.1%) | 15 (14.2%) | 168 (45.4%) | 42 (23.3%) | **593 (36.0%)** |
| **Container Index >1 (%)** | 0 | 0 | 0 | 0 | 0 | 0 | 0 | 0 |
| **Breteau Index >5 (%) **** | 3 (2.0%) | 0 (0.0%) | 0 (0.0%) | 3 (0.7%) | 1 (0.9%) | 7 (1.9%) | 0 (0.0%) | **14 (0.9%)** |
| **Public Sector Reports (%) ***** | 111 (73.5%) | 247 (92.5%) | 136 (82.9%) | 403 (96.9%) | 75 (70.8%) | 363 (97.1%) | 74 (41.4%) | **1,409 (85%)** |
| **Private Sector Reports (%)** | 40 (26.5%) | 20 (7.5%) | 28 (17.1%) | 13 (3.1%) | 31 (29.2%) | 11 (2.9%) | 106 (58.9%) | **249 (15%)** |

**Statistical tests**:
* House Index (HI): χ²(6, N = 1646) = 88.1, *p* < 0.001
** Breteau Index (BI): χ²(6, N = 1646) = 12.4, *p* = 0.053 (not statistically significant)
*** Public vs. Private Reporting: χ²(6, N = 1658) = 405.0, *p* < 0.001

**Note**: Entomological indices represent the proportion of dengue cases from each zone associated with elevated vector indices, defined as HI >1%, CI >1%, and BI >5.
