# Supplementary material for: Spatiotemporal patterns and entomological predictors of dengue transmission in Urban Surat, India (2016–2020): A surveillance-based risk modelling study
Source: PLOS Glob Public Health. 2026 Mar 19;6(3):e0006086. doi: 10.1371/journal.pgph.0006086 (PMC13001960; doi:10.1371/journal.pgph.0006086)
Supplement: Attachment — Submitted filename: Review Report (PLOS Baman Dibeka).pdf [file pgph.0006086.s003.pdf]

Review Report

Reviewer Name: Dr. Dibeka Baman

Journal: Plos Global Public Health

Manuscript Number: PGPH-D-25-01994

Full Title: Spatiotemporal Patterns and Entomological Predictors of Dengue Transmission in Urban Surat, India (2016–2020): A Surveillance-Based Risk Modelling Study

Article Type: Full Length Article

Section-by-Section Analysis

Title

Nothing special

Abstract and Highlights

Adapt the abstract accordingly

Introduction

The background section is up to date and appropriate for the topic. The research gap is identified. However, the problem statement, research question, and hypothesis should have been clearly mentioned.

Methods

The study methods (including applicability/modelling) are reported in sufficient detail to allow for their replicability or reproducibility. However,

- the specific paradigm underpinning the study needs to be added: the post-positivist paradigm with quantitative design
- The study explores the temporal pattern. So, it is important to specify a 'retrospective **longitudinal** design
- Details from lines 103 to 106 don't have anything to do with the materials section. Move it to the introduction section (background)

The statistical analyses, control sampling, mechanism, and statistical reporting are appropriate and well described.

Results

The results presentation is not sufficiently effective to present the study findings.

- From lines 201 to 204, there is a logical issue. " Diagnostic confirmation was achieved through detection of the non-structural protein (NS1) antigen in 66.0% of cases, dengue-specific immunoglobulin M (IgM) antibodies in 44.7%, and both markers in 10.8% of cases."

- In Figure 2: What do you mean by Breatue Index in the legend? Isn't the Breteau Index
- Temporal distribution must successively have at least two presentations of data: the first one is done (annual temporal distribution showing annual trends). The second must be the monthly distribution, showing the seasonal pattern (presenting cumulative data for each month). The description from lines 213 to 216 is not enough to demonstrate the seasonal pattern.
- Details from lines 217 to 220 are the authors' comments, inappropriate in the results section. Please move your comments to the discussion section.
- From lines 224 to 227, please harmonize units (percentage or number). Don't mix them (…in 64% of surveyed areas; however, 12 areas…)
- From lines 231 to 234, do not comment and interpret your results in this section
- In line 259, do not confuse gender with sex

Discussion

The interpretation of results and study conclusions is supported by the data and the study design. However,

- From lines 310 to 312, the rationale is not clear. The high rate reported from the public sector must be interpreted appropriately. Does active surveillance apply both in the public and private sectors? What is the relative size of public versus private health facilities in these areas? The analysis should have taken these elements into account.
- From lines 320 to 323, the argumentation is not logical. The authors showed a weak correlation between vector indices and case counts, while concluding that "Integrating epidemiological and entomological data is therefore essential for early warning and defining actionable thresholds for response
- Delimitations, limitations, and the strengths of the study are poorly described. Please detail factors that undermine the generalizability of the findings (delimitations), the constraints in data interpretation (limitations). Discuss all biases related to your study (selection, measurements, and interpretation biases) to improve transparency.
- References are not recent and diverse enough

Recommendations

<u>Accepted with major improvement</u>

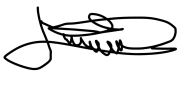

Dr Dibeka Baman
