# Supplementary material for: Spatiotemporal patterns and entomological predictors of dengue transmission in Urban Surat, India (2016–2020): A surveillance-based risk modelling study
Source: PLOS Glob Public Health. 2026 Mar 19;6(3):e0006086. doi: 10.1371/journal.pgph.0006086 (PMC13001960; doi:10.1371/journal.pgph.0006086)
Supplement: Attachment — Submitted filename: Response to Reviewers and Journal requirnment.docx [file pgph.0006086.s005.docx]

RESPONSE TO REVIEWERS

We sincerely thank the Editors and Reviewers for their thorough evaluation of our manuscript and for the constructive, insightful comments. These suggestions have substantially enhanced the clarity, methodological rigor, interpretability, and policy relevance of our work. Below, we provide a consolidated, point-by-point response to each reviewer.

Two files have been submitted:

1. **Revised Manuscript with Track Changes** – showing all modifications.
2. **Unmarked Version – labelled “Manuscript”** – the final clean version of the revised manuscript.

Line numbers are provided where applicable in both files for ease of reference.

**REVIEWER #1**

**Reviewer Comment 1:** The logistic regression model predicts moderate-to-high vector density (House Index ≥1%), which is programmatically relevant. However, the manuscript would benefit from a clearer explanation of why this outcome was selected and how the model is intended to be used operationally (e.g., early warning, prioritization of vector control activities, or surveillance planning).

**Response:** We selected House Index (HI) ≥1% based on National Vector Borne Disease Control Programme (NVBDCP) guidelines, which define elevated dengue risk as HI ≥1%, Container Index (CI) ≥10%, and Breteau Index (BI) ≥5%. These indices are routinely used by NVBDCP to monitor Aedes aegypti, the primary container-breeding vector of dengue. Monthly entomological surveillance, conducted by the Surat Municipal Corporation’s sentinel system, measured HI, CI, and BI across administrative zones. The logistic regression model serves as a decision-support tool to guide the operational prioritization of vector control interventions and surveillance planning.
*(Revised Manuscript with Track Changes Line no-179; Unmarked Manuscript Line no-162)*

**Reviewer Comment 2:** The observed decline in dengue incidence over time—particularly during 2019–2020—is likely influenced by underreporting associated with COVID-19–related disruptions. I recommend consistently referring to these trends as “reported dengue incidence” and more explicitly emphasizing the role of surveillance artefacts when interpreting temporal patterns.

**Response:** We clarified that the observed decline reflects reported dengue incidence and surveillance artefacts rather than actual transmission intensity. Reported incidence decreased from 11.1 to 2.2 per 100,000 population, diverging from national trends. The pronounced drop in 2019–2020 likely reflects COVID-19–related underreporting due to reduced healthcare access and diversion of resources.
*(Revised Manuscript with Track Changes Line no-381; Unmarked Manuscript Line no-320)*

**Reviewer Comment 3:** The model demonstrates moderate discrimination with high specificity but relatively low sensitivity and limited explanatory power. Framing the model as a screening or prioritization tool may help avoid overinterpretation.

**Response:** The model demonstrated moderate discrimination (AUC = 0.687), high specificity (87.3%), and low sensitivity (35.9%). It should be interpreted as a screening or prioritization tool for identifying lower-risk areas, rather than as a precise predictor of dengue hotspots.
*(Revised Manuscript with Track Changes Line no-464; Unmarked Manuscript Line no-377)*

**Reviewer Comment 4:** The discussion appropriately notes the weak and inconsistent relationship between larval indices and dengue incidence. This section could be strengthened by further acknowledging the limitations of larval indices as proxies for transmission risk, particularly in the absence of adult mosquito density measures, climatic variables, or human mobility data.

**Response:** We clarified that larval indices (HI, CI, BI) are limited predictors of dengue risk. They indicate vector presence but do not capture adult mosquito density, vector competence, human–vector contact, or viral circulation. In the absence of adult vector, climatic, and mobility data, larval indices should be interpreted as indicators of vector presence rather than transmission intensity. Integrated epidemiological and entomological surveillance remains essential for situational awareness and preparedness, rather than precise outbreak prediction.
*(Revised Manuscript with Track Changes Line no-432; Unmarked Manuscript Line no-364)*

**Reviewer Comment 5:** The finding of substantial underreporting from private healthcare facilities is important. Additional discussion on possible contributing factors and implications for strengthening integrated urban surveillance would add value.

**Response:** We clarified that underreporting from private healthcare facilities remains a critical gap, driven by variable compliance, limited diagnostic confirmation, administrative burden, and lack of incentives. Strengthening urban dengue surveillance requires mandatory reporting across all sectors, interoperable digital systems, and ongoing engagement and training of private practitioners.
*(Revised Manuscript with Track Changes Line no-396; Unmarked Manuscript Line no-327)*

**Reviewer Comment 6:** Minor language and stylistic edits—particularly in the Discussion—would improve clarity and flow but do not detract from the overall quality of the manuscript.

**Response:** Language and stylistic edits were made throughout the Discussion to improve clarity and readability without altering content.

**REVIEWER #2**

**Title**
**Reviewer Comment:** Nothing special.
**Response:** No changes were requested; the title has been retained.

**Abstract**
**Reviewer Comment:** Adapt the abstract accordingly.
**Response:** The abstract has been comprehensively revised to reflect the clarified problem statement, study design, analytical approach, key results, and applied public health implications. It is now fully aligned with the updated manuscript content.

**Introduction**
**Reviewer Comment:** The background is appropriate and up to date; however, the problem statement, research question, and hypothesis should be clearly stated.
**Response:** An explicit problem statement, research question, and hypothesis have been added to the Introduction to clearly articulate the study objectives and address the identified research gap.
*(Revised Manuscript with Track Changes Line no-114; Unmarked Manuscript Line no-109)*

**Methods**
**Reviewer Comment 1:** The paradigm underpinning the study should be specified.
**Reviewer Comment 2:** The temporal nature of the study requires specification of a retrospective longitudinal design.
**Response:** The Methods section now explicitly states that the study follows a post-positivist paradigm with a quantitative, retrospective longitudinal design, utilizing cross-sectional programmatic surveillance data from Surat.
*(Revised Manuscript with Track Changes Line no-148; Unmarked Manuscript Line no-134)*

**Reviewer Comment:** Details from lines 103–106 do not belong in the Materials section and should be moved to the Introduction.
**Response:** Climate-related contextual information previously included in the Materials section has been relocated to the Introduction to better frame the study background.

**Results**
**Reviewer Comment 1:** The results presentation is not sufficiently effective.
**Response:** The Results section has been restructured for clarity and coherence, with improved organization of tables, figures, and narrative descriptions.

**Reviewer Comment 2:** Lines 201–204 contain a logical issue regarding diagnostic confirmation.
**Response:** The wording describing diagnostic confirmation has been clarified to remove logical ambiguity and improve interpretability.
*(Revised Manuscript with Track Changes Line no-266; Unmarked Manuscript Line no-227)*

**Reviewer Comment 3:** Figure 2 legend refers to “Breatue Index” instead of “Breteau Index.”
**Response:** This typographical error has been corrected.

**Reviewer Comment 4:** Temporal analysis should include both annual and cumulative monthly distributions to demonstrate seasonality.
**Response:** The temporal analysis has been strengthened by presenting both annual trends and cumulative monthly distributions, allowing clearer interpretation of seasonal patterns.
*(Revised Manuscript with Track Changes Line no-275; Unmarked Manuscript Line no-236)*

**Reviewer Comment 5:** Author comments and interpretation appear in the Results section (lines 217–220 and 231–234).
**Response:** All interpretative commentary has been removed from the Results section and relocated to the Discussion to maintain appropriate section boundaries.

**Reviewer Comment 6:** Units are inconsistently reported (percentages and absolute numbers mixed).
**Response:** Units have been harmonized throughout the Results section. Percentages are used consistently, with absolute counts reported only when necessary and clearly labelled.

**Reviewer Comment 7:** Line 259 confuses gender with sex.
**Response:** Terminology has been corrected.
*(Revised Manuscript with Track Changes Line no-326; Unmarked Manuscript Line no-270)*

**Discussion**
**Reviewer Comment 1:** The rationale regarding higher reporting from the public sector is unclear.
**Response:** The Discussion now clarifies that active surveillance is primarily implemented through public-sector infrastructure. Consequently, higher reporting reflects the surveillance system design rather than healthcare utilization patterns across public and private sectors.
*(Revised Manuscript with Track Changes Line no-395; Unmarked Manuscript Line no-328)*

**Reviewer Comment 2:** The argument regarding weak correlations between vector indices and dengue incidence is not logically developed.
**Response:** This section has been revised to clarify that weak correlations do not undermine the value of integrated surveillance. Rather, they highlight the limitations of relying on single data streams. The revised discussion emphasizes that integration of epidemiological and entomological data improves situational awareness and preparedness, rather than precise outbreak prediction, in line with WHO guidance.
*(Revised Manuscript with Track Changes Line no-436; Unmarked Manuscript Line no-376)*

**Reviewer Comment 3:** Delimitations, limitations, strengths, and biases are poorly described.
**Response:** The manuscript has been substantially revised to expand the sections on delimitations, limitations, and strengths. Factors affecting generalizability, data constraints, and potential selection, measurement, and interpretation biases are now explicitly discussed to enhance transparency.

**References**
**Reviewer Comment:** References are not sufficiently recent or diverse.
**Response:** The reference list has been updated to include more recent and diverse literature (2019–2025), covering dengue surveillance, vector ecology, urban public health, and modeling approaches.

**REVIEWER #3**

**Abstract**
**Reviewer Comment 1:** The abstract should include the objective of the study at the end of the background section.
**Response:** The study objective has been explicitly added at the end of the Background subsection of the abstract to clearly state the purpose of the study.

**Reviewer Comment 2:** The term “retrospective analysis” (line 11) is unclear and may refer to either study design or data collection.
**Response:** The abstract and Methods section have been revised to clearly define “retrospective analysis” as a retrospective longitudinal study design using existing surveillance data, explicitly linking the term to study design rather than data collection.

**Methods**
**Reviewer Comment 1:** The Methods section should clearly describe the study design, population, period, area, data collection process, tools, and software used. Data extraction procedures are unclear.
**Response:** The Methods section has been comprehensively revised to serve as a clear methodological blueprint. It now explicitly specifies:

- **Study design:** Quantitative, retrospective longitudinal study
- **Study population:** All laboratory-confirmed dengue cases
- **Study period:** January 2016–December 2020
- **Study area:** Surat, Gujarat, India
- **Data source:** Surat Municipal Corporation (SMC) Central Sentinel Surveillance database
- **Data extraction:** Surveillance data on 1,658 laboratory-confirmed dengue cases were retrieved from the SMC database with formal administrative approval
- **Analytical approach:** Descriptive analysis, chi-square test, and binomial logistic regression
- **Software:** Statistical software Jamovi used for data entry, management, and analysis is specified in the revised Methods section

These revisions enhance transparency and reproducibility.

**Reviewer Comment 2:** How were associated factors identified, and how were multicollinearity assumptions checked?
**Response:** This has been clarified in the Methods section. A binomial logistic regression model was used to identify predictors of moderate-to-high vector density (HI ≥1%), consistent with NVBDCP risk thresholds. Areas were classified as low-risk (HI <1%) or moderate-to-high risk (HI ≥1%).

Candidate predictor variables included age (continuous), sex, year, month, and residential zone (categorical). Multicollinearity was assessed using Pearson’s correlation coefficients, variance inflation factors (VIF range: 1.01–1.2), and tolerance values, confirming the absence of significant multicollinearity.
*(Revised Manuscript with Track Changes Line no-249; Unmarked Manuscript Line no-213)*

**Results and Conclusions**
**Reviewer Comment 1:** How were candidate variables selected for multivariable logistic regression? How was persistent vector presence assessed? Conclusions and recommendations are not aligned with the results.
**Response:**
A binomial logistic regression model was used to identify predictors of moderate-to-high vector density (HI ≥1%) in accordance with NVBDCP risk thresholds. Areas were classified as low-risk (HI <1%) or moderate-to-high risk (HI ≥1%). Predictor variables included age (continuous), sex, year, month, and residential zone (categorical).

Persistent vector presence was inferred through repeated monthly exceedance of standard larval index thresholds across administrative zones, rather than direct measurement of entomological persistence.

The Conclusion and Recommendations sections have been revised to ensure that all statements are directly supported by the reported results and the surveillance context, avoiding over-interpretation.

**Main Document**

**Introduction**
**Reviewer Comment:** Lines 86–87 contain an uncited statement; the magnitude and consequences of the problem should be better supported.
**Response:** The uncited statement has been removed. The Introduction has been streamlined and strengthened with appropriate citations to better reflect the magnitude, severity, and public health consequences of dengue.

**Methods (Main Document)**
**Reviewer Comment 1:** The study design and period are unclear, and figures do not follow standard format.
**Response:** The study design and period are now clearly stated at the beginning of the Methods section. All figures have been revised and reformatted to follow standard scientific conventions.
*(Revised Manuscript with Track Changes Line no-148; Unmarked Manuscript Line no-134)*

**Reviewer Comment 2:** Why use chi-square tests if logistic regression was performed?
**Response:** Chi-square tests were retained as exploratory bivariate analyses to assess crude associations between categorical variables and outcomes. Binomial logistic regression was subsequently used to identify independent predictors of moderate-to-high vector density (HI ≥1%).
*(Revised Manuscript with Track Changes Line no-246)*

**Results**
**Reviewer Comment 1:** Figure captions are incorrectly placed above figures. Age analysis is unclear.
**Response:** All figure captions have been relocated below the figures, in accordance with journal guidelines. Age has been consistently analyzed and reported as a continuous variable.
*(Revised Manuscript with Track Changes Line no-249; Unmarked Manuscript Line no-213)*

**Discussion**
**Reviewer Comment:** The Discussion is shallow and does not follow a standard scientific structure.
**Response:** The Discussion section has been substantially expanded and reorganized to follow a standard scientific framework, including:

- Interpretation of key findings in relation to study objectives
- Comparison with existing literature
- Public health and programmatic implications
- Methodological strengths and limitations

All interpretations are now explicitly grounded in the study findings and the surveillance context.

**Conclusion and Recommendations**
**Reviewer Comment:** These sections were missing.
**Response:** A dedicated Conclusion and Recommendations section has been added. This section summarizes the key findings and provides evidence-based, programmatically relevant recommendations that are fully aligned with the study results.

**REVIEWER #4**

**Abstract**
**Reviewer Comment 1:** The implications for dengue control could be made more actionable, particularly in terms of targeted interventions.
**Response:** The abstract has been revised to explicitly emphasize actionable implications for dengue control, including targeted vector control interventions, prioritization of high-risk zones, and integration of epidemiological and entomological surveillance to support urban public health decision-making.

**Introduction**
**Reviewer Comment 2:** The role of urbanization and climate change in exacerbating dengue risk could be framed more strongly within global health policy contexts.
**Response:** The Introduction has been strengthened to explicitly link unplanned urban expansion, inadequate water management, high human mobility, and climate variability with increasing urban dengue risk. Dengue prevention is now situated within broader global health priorities, including climate resilience, sustainable urban development, and equitable health systems.
*(Revised Manuscript with Track Changes Line no-84; Unmarked Manuscript Line no-79)*

**Discussion**
**Reviewer Comment 3:** Further context is needed on the limitations of vector indices for predicting dengue incidence.
**Response:** The Discussion now explicitly describes the limitations of larval indices (House Index, Container Index, Breteau Index). These indices do not capture adult mosquito density, vector competence, human–vector contact, or viral circulation. In the absence of adult mosquito surveillance, climatic variables, and human mobility data, larval indices are now clearly interpreted as indicators of vector presence rather than transmission intensity.
*(Revised Manuscript with Track Changes Line no-428; Unmarked Manuscript Line no-359)*

**Reviewer Comment 4:** Clarify the choice of logistic regression over more complex spatiotemporal or Bayesian approaches.
**Response:** The rationale for using binomial logistic regression has been clarified. This approach was selected due to its alignment with NVBDCP operational thresholds (HI ≥1%), interpretability, and suitability for programmatic decision-making. The binary classification of areas into low-risk and moderate-to-high risk supports actionable public health planning, while acknowledging that more complex models may better capture spatial autocorrelation and temporal dependencies.
*(Revised Manuscript with Track Changes Line no-246)*

**Reviewer Comment 5:** Underreporting by private providers should be explored in more detail.
**Response:** The Discussion now expands on surveillance gaps related to the private sector, including variable reporting compliance, limited laboratory confirmation, administrative burden, lack of incentives, and weak enforcement of notification mandates, with further deterioration during the COVID-19 pandemic. The predominance of public-sector reporting is explicitly framed as a surveillance system design issue, and policy-relevant strategies to strengthen public–private surveillance integration are outlined.
*(Revised Manuscript with Track Changes Line no-395; Unmarked Manuscript Line no-327)*

**Reviewer Comment 6:** Further discussion is needed on the model’s low sensitivity despite high specificity.
**Response:** The Discussion now explicitly explains that the model’s low sensitivity (35.9%), despite high specificity (87.3%), reflects the omission of key determinants of dengue risk, including climatic variability, housing and water storage practices, population mobility, and socioeconomic factors such as migration and urban poverty. The model is therefore framed as a screening and prioritization tool, better suited for identifying lower-risk areas than for precise hotspot prediction.
*(Revised Manuscript with Track Changes Line no-458; Unmarked Manuscript Line no-371)*

**Reviewer Comment 7:** Greater emphasis on incorporating socioeconomic factors into predictive models would strengthen the conclusions.
**Response:** The Discussion now explicitly recommends incorporating socioeconomic variables (e.g., migration patterns, urban poverty), alongside climatic and mobility data, into future predictive models to enhance dengue control and urban health planning.
*(Revised Manuscript with Track Changes Line no-458; Unmarked Manuscript Line no-371)*

**Conclusion**
**Reviewer Comment 8:** The Conclusion could be more actionable by outlining specific dengue control strategies.
**Response:** The Conclusion has been revised to include concrete, actionable dengue control strategies, such as risk-based vector control deployment, enhanced surveillance in persistent-risk zones, and strengthened public–private reporting mechanisms to improve urban dengue preparedness and response.

**RESPONSE TO JOURNAL REQUIREMENTS**

**1. Ethics Statement Placement**
**Comment:** Please ensure that your Ethics Statement is available in its entirety at the beginning of your Methods section, under a subheading “Ethics Statement.”
**Response:** The Ethics Statement has been relocated to the beginning of the Methods section under the subheading **“Ethics Statement”** in the revised manuscript. Ethical approval was obtained from the Institutional Ethics Committee of Government Medical College, Surat (Approval No. 7319/21, dated 04 August 2021), with administrative clearance from the Surat Municipal Corporation. As this study involved secondary analysis of anonymized dengue surveillance data, the requirement for individual informed consent was waived.

**2. Figure File Requirements**
**Comment:** Please upload separate figure files in .tif or .eps format and remove the figures from the manuscript file while retaining the legends.
**Response:** All figures have been removed from the main manuscript, and only figure legends have been retained. Each figure has been uploaded separately in compliant **.TIF** format. The figure files were verified using PLOS’s NAAS tool, and only compliant versions have been submitted.

**3. Supporting Information Legends**
**Comment:** Supporting Information files were uploaded, but a list of legends was not included.
**Response:** A complete list of legends for all Supporting Information files has now been added after the References section in the revised manuscript.

**4. Data Availability Statement and Data Sharing**
**Comment:** Please clarify your data availability plan and ensure compliance with PLOS’s open data policy, or revise the statement with justification if full public deposition is not possible.
**Response:** The dengue surveillance dataset used in this study was systematically collected by the Surat Municipal Corporation (SMC) since 2007. For this retrospective analysis, data on 1,658 **laboratory-confirmed reported dengue cases** (January 2016–December 2020) were retrieved from the SMC Central Sentinel Surveillance database with administrative permission from the Health Commissioner. The dataset was accessed on 25 December 2021.

In accordance with PLOS data-sharing policies, the anonymized dataset will be made publicly available upon acceptance of the manuscript, subject to institutional approval and without any personal identifiers, ensuring compliance with the ethics committee–approved protocol.

**5. Copyright and Licensing of Figures and Maps**
**Comment:** Please clarify copyright status and licensing for figures and maps, including map base layers and shapefiles, and ensure compatibility with the CC BY 4.0 license.
**Response:** The manuscript includes six figures:

- **Figures 1, 2, 4, and 5 (Analytical charts):** Original analytical charts generated by the authors using study data. No third-party or copyrighted material was used. The authors retain full copyright and confirm these figures may be published under **CC BY 4.0**.
- **Figure 3 (Spot map):** Generated by the authors using ArcGIS Online with patient latitude and longitude coordinates. The base shapefile for Surat Municipal Corporation boundaries was obtained from **DIVA-GIS** (<https://www.diva-gis.org/gdata>), licensed under a CC BY 4.0–compatible open license. The source and license are stated in the figure legend. No proprietary mapping services (e.g., Google Maps) were used. The shapefile is also provided as a Supporting Information file for transparency and reproducibility.
- **Figure 6 (Time-trend analysis):** Presents a historical time-trend analysis using publicly available, aggregated SMC dengue surveillance data. All data are non-identifiable and open-source. The figure was independently generated by the authors and does not reproduce copyrighted material.
