# Supplementary material for: Spatiotemporal patterns and entomological predictors of dengue transmission in Urban Surat, India (2016–2020): A surveillance-based risk modelling study
Source: PLOS Glob Public Health. 2026 Mar 19;6(3):e0006086. doi: 10.1371/journal.pgph.0006086 (PMC13001960; doi:10.1371/journal.pgph.0006086)
Supplement: Attachment — Submitted filename: Reviewer Comments.docx [file pgph.0006086.s002.docx]

**Reviewer Comments**

**Manuscript ID:** PGPH-D-25-01994
**Title:** *Spatiotemporal Patterns and Entomological Predictors of Dengue Transmission in Urban Surat, India (2016–2020)*

**General Comments**

I appreciate the opportunity to review this manuscript. The authors present a thoughtful and well-executed analysis of five years of dengue surveillance and entomological data from an important urban setting in India. Integrating laboratory-confirmed dengue cases with routine vector surveillance at city scale is a notable strength of this work and highly relevant to public health practice in dengue-endemic, rapidly urbanizing contexts.

Overall, the manuscript is clearly written, methodologically sound, and well aligned with the scope of *PLOS Global Public Health*. The findings—particularly the persistence of vector indices despite declining reported dengue incidence and the identification of spatial hotspots—are valuable and policy-relevant. That said, several conceptual and interpretive issues require clarification to strengthen the manuscript and ensure that readers fully understand the public health implications of the analyses.

I recommend **major revision**, primarily to improve clarity around the modelling rationale, interpretation of surveillance trends, and operational relevance of the findings.

**Major Comments**

**1. Clarifying the public health purpose of the regression model**

The logistic regression model predicts moderate-to-high vector density (HI ≥1%) rather than dengue incidence or outbreak risk. While this outcome is programmatically meaningful, the manuscript would benefit from a clearer explanation of **why predicting vector density is the chosen endpoint** and how this model would be used in practice by municipal or surveillance teams.

I suggest the authors explicitly state:

- How this model could inform early warning, prioritization of vector control, or resource allocation
- Whether the model is intended to complement routine entomological surveillance or to fill specific operational gaps

Clarifying this will help readers better appreciate the applied value of the modelling work.

**2. Interpretation of declining dengue incidence**

The observed decline in reported dengue incidence from 2016 to 2020 is interesting but difficult to interpret given acknowledged underreporting, especially during the COVID-19 period. While the Discussion mentions pandemic-related disruptions, the Results still present the trend in a way that could be interpreted as a true epidemiological decline.

I recommend:

- Consistently referring to this as **“reported dengue incidence”**
- More clearly emphasizing that trends—particularly in 2019–2020—likely reflect surveillance artefacts rather than reduced transmission
- Considering a brief comparison between pre-COVID (2016–2018) and pandemic-affected years to contextualize the findings

**3. Limited explanatory power of the model**

The regression model shows moderate discrimination (AUC ~0.69) but low sensitivity and explains a relatively small proportion of variance. While this is acknowledged, the implications could be discussed more explicitly.

It may be helpful to frame the model as:

- Better suited for **ruling out low-risk areas** rather than precisely identifying hotspots
- A screening or prioritization tool rather than a predictive outbreak model

This framing would align well with the results and avoid overinterpretation.

**4. Entomological indices and transmission dynamics**

The manuscript appropriately notes the weak and inconsistent relationship between larval indices and dengue incidence. This is an important finding. However, the Discussion could be strengthened by more clearly acknowledging the limitations of larval indices as proxies for transmission risk, particularly in the absence of:

- Adult mosquito density measures
- Climatic variables
- Human mobility or socioeconomic factors

This would further contextualize the modest performance of the predictive model.

**5. Private-sector underreporting**

The observation that public-sector facilities reported substantially more cases than private providers is important and well documented in the Results. The Discussion would benefit from a deeper reflection on possible explanations—such as reporting compliance, diagnostic access, or incentives—and clearer articulation of the implications for strengthening urban dengue surveillance systems.

**Minor Comments**

1. Please ensure consistent use of terminology, particularly “reported dengue cases/incidence.”
2. Some figures would benefit from more descriptive legends to allow interpretation without referring back to the text.
3. Statistical reporting could be made more consistent by including test statistics alongside p-values where feasible.
4. Minor language and stylistic edits—especially in the Discussion—would improve readability and flow.

**Strengths**

- Use of laboratory-confirmed dengue cases
- Integration of epidemiological, entomological, temporal, and spatial data
- Five-year city-wide dataset with clear operational relevance
- Honest acknowledgment of surveillance limitations
- Strong potential to inform urban vector control strategies

**Conclusion**

This is a solid and policy-relevant contribution to the dengue surveillance literature. With clearer articulation of the modelling rationale, more cautious interpretation of incidence trends, and strengthened discussion of operational implications, the manuscript would be suitable for publication in *PLOS Global Public Health*.

**Recommendation**

This manuscript addresses an important and timely public health issue and is based on a solid surveillance dataset integrating epidemiological, entomological, and spatial information. The analyses are generally appropriate, and the findings are relevant for urban dengue control in endemic settings.

While the study has clear strengths and good potential for publication, several aspects—particularly the framing and interpretation of the regression model, the discussion of declining reported incidence, and the articulation of public health implications—would benefit from clarification and refinement.

I therefore recommend **publication after major revision**. With thoughtful revisions addressing the points raised, this manuscript would make a valuable contribution to the literature and be well suited for *PLOS Global Public Health*.
